# P-767. Common uropathogens identified in patients with non-binary gender

**DOI:** 10.1093/ofid/ofaf695.978

**Published:** 2026-01-11

**Authors:** Roxanna Mohammed, Eugene Yeung

**Affiliations:** University of British Columbia, Vancouver, British Columbia, Canada; University of British Columbia, Vancouver, British Columbia, Canada

## Abstract

**Background:**

Not much is known about the common uropathogens in patients with non-binary gender. Their prevalence and distribution of uropathogens could be different from patients identified as males or females. It is unknown whether the empiric antimicrobial therapy for males and females with urinary tract infections apply to non-binary patients. An audit was conducted to investigate the distribution of common microorganisms reported in urine culture of non-binary patients in British Columbia (BC), Canada.

**Methods:**

LifeLabs BC microbiology laboratories, connected with 129 collection centres in urban and rural communities in the province, provided the laboratory data for urinary culture (n = 1597489) of male, female, and non-binary patients from October 31, 2019 to September 30, 2024, of which 273777 specimens showed significant growth. Gender was identified from the information provided on laboratory requisition forms completed by healthcare providers.

**Results:**

Among the non-binary patients, 33 of the 120 urine specimens (27.5%) showed significant growth, compared to 273744 of the 1597369 urine specimens (17.1%) from the male and female patients combined (p < 0.05). Only 3 of the non-binary patients were infants (< 1-year-old). *Escherichia coli* (n = 20), *Enterococcus faecalis* (n = 4), *Klebsiella pneumoniae* (n = 4), *Staphylococcus saprophyticus* (n = 3), and *Streptococcus agalactiae* (n = 2) were the most common uropathogens identified. Antimicrobial susceptibility testing was reported on 19 *E. coli* isolates of the non-binary patients: cefazolin 100% susceptible, ciprofloxacin 63% susceptible, gentamicin 100% susceptible, meropenem 100% susceptible, nitrofurantoin 95% susceptible, and sulfamethoxazole-trimethoprim 74% susceptible.

**Conclusion:**

*E. coli* is the most common uropathogen among non-binary patients, like male and female patients,. Further investigations are needed to determine why urine culture positivity rate was significantly higher in non-binary patients.

**Disclosures:**

All Authors: No reported disclosures

